# Supplementary material for: Revealing the tissue-level complexity of endogenous glucagon-like peptide-1 receptor expression and signaling
Source: Nat Commun. 2023 Jan 18;14:301. doi: 10.1038/s41467-022-35716-1 (PMC9849236; doi:10.1038/s41467-022-35716-1)
Supplement: Supplementary file 1 — Supplementary Information [file 41467_2022_35716_MOESM1_ESM.pdf]

SUPPLEMENTARY INFORMATION

Revealing the tissue-level complexity of endogenous glucagon-like peptide-1 receptor expression and signaling

Julia Ast[1], Daniela Nasteska[1], Nicholas H.F. Fine[1], Daniel J. Nieves[2], Zsombor Koszegi[1], Yann Lanoiselée[1], Federica Cuozzo[1], Katrina Viloria[1], Andrea Bacon[3], Nguyet T. Luu[4,5], Philip N. Newsome[4,5], Davide Calebiro[1], Dylan M. Owen[2], Johannes Broichhagen[6*] and David J. Hodson[1,7*]

[1] Institute of Metabolism and Systems Research (IMSR), and Centre of Membrane Proteins and Receptors (COMPARE), University of Birmingham, Birmingham, UK.
[2]Institute for Immunology and Immunotherapy, and Centre of Membrane Proteins and Receptors (COMPARE), University of Birmingham, Birmingham, UK.
[3] Genome Editing Facility, Technology Hub, University of Birmingham, Birmingham, UK
[4] National Institute for Health Research Biomedical Research Centre at University Hospitals Birmingham NHS Foundation Trust, University of Birmingham, Birmingham, United Kingdom.
[5] Centre for Liver and Gastrointestinal Research, Institute of Immunology and Immunotherapy, University of Birmingham, Birmingham, United Kingdom.
[6] Leibniz-Forschungsinstitut für Molekulare Pharmakologie, Berlin, Germany.
[7] Oxford Centre for Diabetes, Endocrinology and Metabolism (OCDEM), NIHR Oxford Biomedical Research Centre, Churchill Hospital, Radcliffe Department of Medicine, University of Oxford, Oxford, OX3 7LE, UK.

*Correspondence should be addressed to:
david.hodson@ocdem.ox.ac.uk, broichhagen@fmp-berlin.de

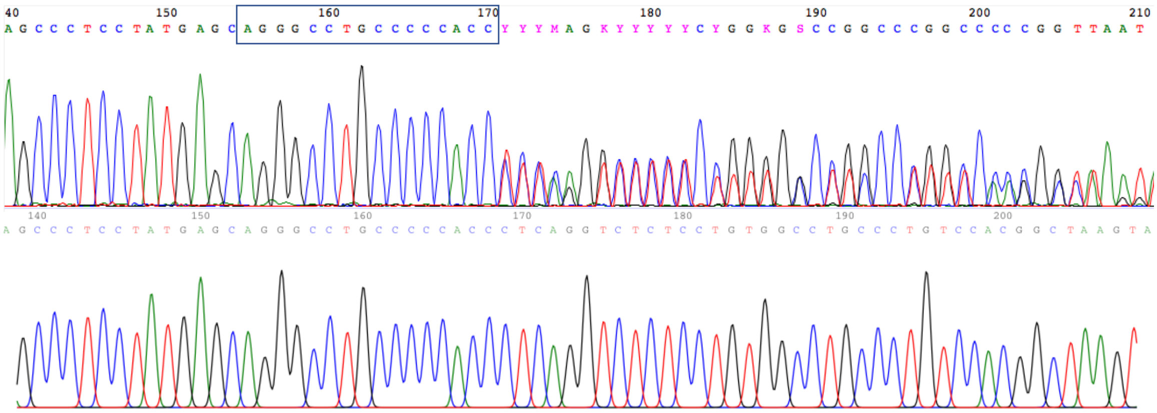

Supplementary Figure 1: Sequencing reveals mutation in potential off-target site in founder. The region around the predicted off-target site within an intron of gene NM_030721 was amplified via PCR using the primers 5'-CTCTGACCCGTGTGTTACCT-3' and 5'-GCATGGATACCACTCCCACC-3' and analyzed via Sanger sequencing. The guide sequence is partly highlighted in the box and indels were introduced close to the Cas9 cut site in the founder mouse (top). Only offspring without the mutation (bottom) were taken forward for breeding.

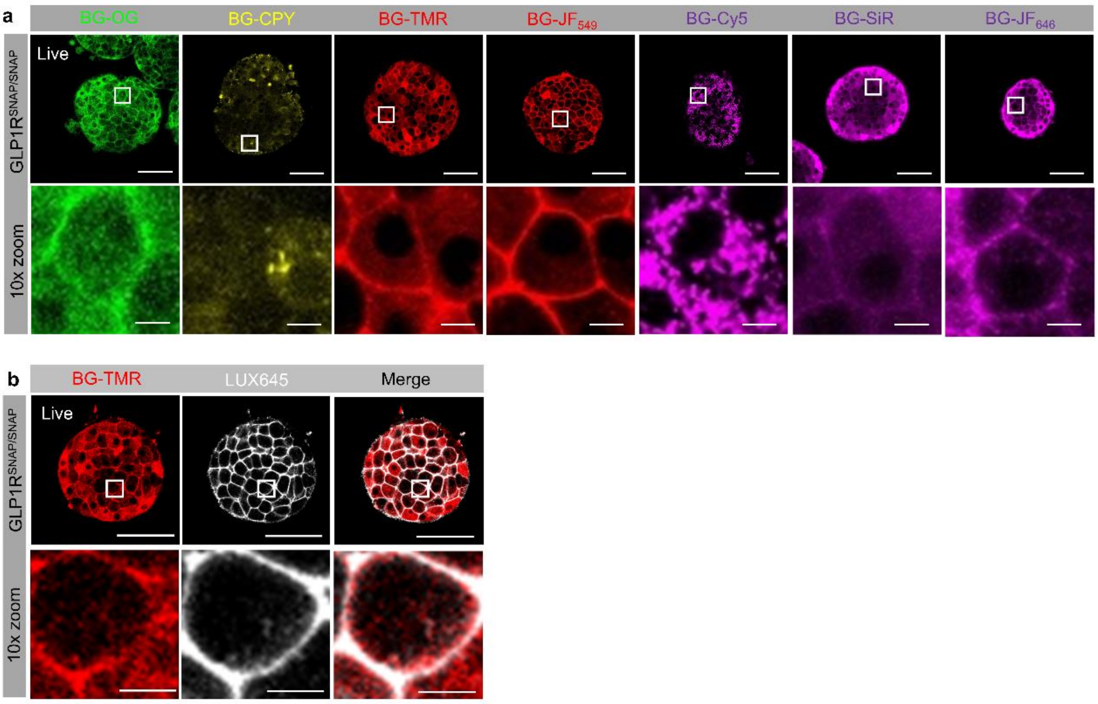

Supplementary Figure 2: SNAP labeling in intact and dissociated GLP1R$^{SNAP/SNAP}$ islets. a) Magnifications of Figure 2b showing single cell GLP1R$^{SNAP/SNAP}$ labeling with BG-OG, BG-CPY, BG-TMR, BG-JF$_{549}$, BG-Cy5, BG-SiR and BG-JF$_{646}$ (n = 16-42 islets, 3-7 animals) (scale bar = 85 µm, except for zoom in scale bar = 8.5 µm). b) Zoomed image from Figure 2e showing co-localization of BG-TMR and LUXendin645 (LUX645) labeling in single GLP1R$^{SNAP/SNAP}$ cells (n = 17 islets, 4 animals) (scale bar = 85 µm for all images, except for zoom in scale bar = 8.5 µm).

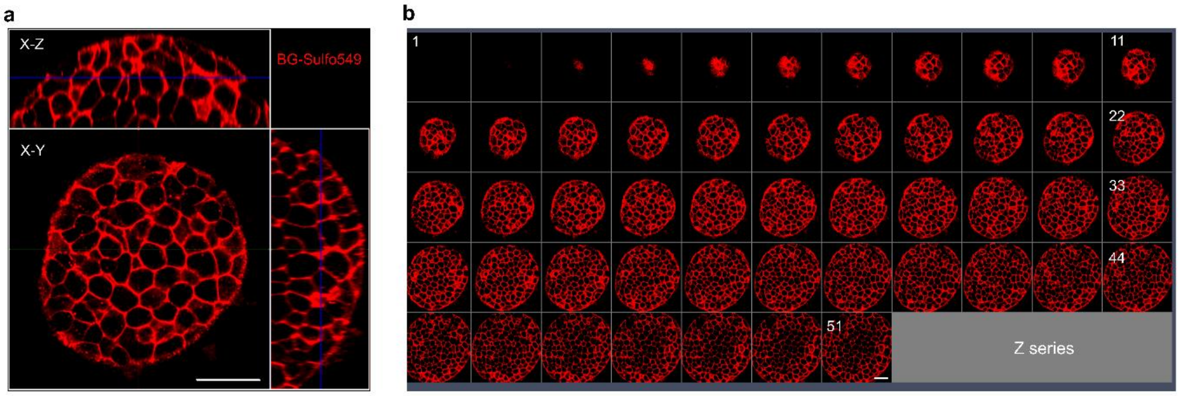

Supplementary Figure 3: Fluorescent labelling is detected at depth within $GLP1R^{SNAP/SNAP}$ islets. a and b) Confocal z-stack (a) and z-series (b) showing intense BG-Sulfo549 labelling within the 5th islet cell layer located at 50 µm depth within the islet (n = 24 islets, 11 animals) (Scale bar = 34 µm).

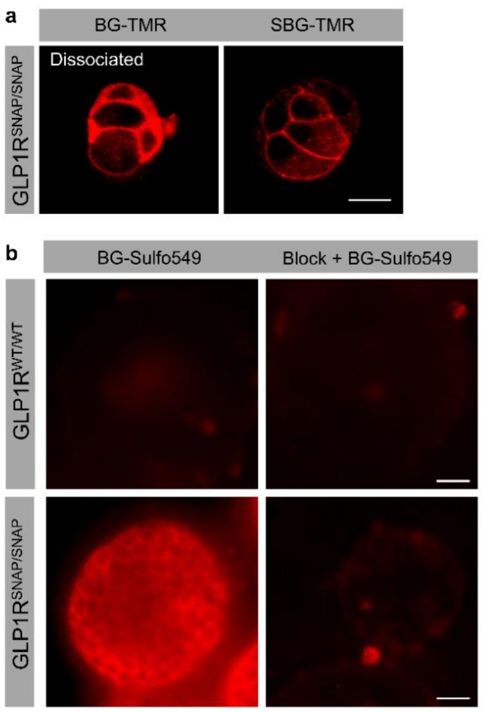

Supplementary Figure 4: Tissue-dependency and specificity of sulfonated SNAP labels. a) Sulfonation of BG-TMR to produce SBG-TMR leads to membrane-restricted GLP1R$^{SNAP/SNAP}$ labelling in dissociated beta cells (~150 islets per animal, n = 4 animals) (scale bar = 11 µm). b) BG-Sulfo549 labelling can be blocked by prior application of BG-Block in GLP1R$^{SNAP/SNAP}$ islets (n = 25-33 islets, 5 animals) (scale bar = 30 µm).

Supplementary Table 1: Selected predicted off-targets for guide sequence AGGGCCGGCCCCCGCCCUC.

Sequence	Mismatches	UCSC gene	Locus
AGGGCCCGGCCCCGCCCTCCAG	2MMs [7:9]		chr9:-114887514
AGGGCCTGCCCCCACCCTCAGG	2MMs [7:14]	NM_030721	chr5:-35931971
AGGGCACGGCCCCGCCCTCTGG	3MMs [6:7:9]	NM_001081023	chr1:-138015670
GGGTGCGGCCCCCGCCCTCAGG	3MMs [1:4:5]	NM_011599	chr4:+71861176
ACTCCCTGCCCCCGCCCTCTAG	4MMs [2:3:4:7]		chr4:+53051322
AGCGCCAGGCCCCGCCCTCTGG	3MMs [3:7:9]	NM_144784	chr9:-53418526
GAGGCCGGGCCCCGCCCTCTGG	3MMs [1:2:9]		chr5:+113593900
GCCGCCGGTCCCCGCCCTCGGG	4MMs [1:2:3:9]	NM_028194	chr5:+73647675
AGGGCCAGACCCCGCCCACTGG	3MMs [7:9:18]	NM_029834	chr7:+4434126
GGGCCCCGCACCCGCCCTCCGG	4MMs [1:4:7:10]	NM_029688	chr2:-151931498
