# Supplementary material for: Revealing the tissue-level complexity of endogenous glucagon-like peptide-1 receptor expression and signaling
Source: Nat Commun. 2023 Jan 18;14:301. doi: 10.1038/s41467-022-35716-1 (PMC9849236; doi:10.1038/s41467-022-35716-1)
Supplement: Supplementary file 2 — Reporting Summary [file 41467_2022_35716_MOESM2_ESM.pdf]

nature portfolio

Corresponding author(s): Johannes Broichhagen, David J. Hodson

Last updated by author(s): Dec 15, 2022

Reporting Summary

Nature Portfolio wishes to improve the reproducibility of the work that we publish. This form provides structure for consistency and transparency in reporting. For further information on Nature Portfolio policies, see our Editorial Policies and the Editorial Policy Checklist.

Statistics

For all statistical analyses, confirm that the following items are present in the figure legend, table legend, main text, or Methods section.

n/a	Confirmed	
☐	☒	The exact sample size (n) for each experimental group/condition, given as a discrete number and unit of measurement
☐	☒	A statement on whether measurements were taken from distinct samples or whether the same sample was measured repeatedly
☐	☒	The statistical test(s) used AND whether they are one- or two-sided<br>*Only common tests should be described solely by name; describe more complex techniques in the Methods section.*
☒	☐	A description of all covariates tested
☐	☒	A description of any assumptions or corrections, such as tests of normality and adjustment for multiple comparisons
☐	☒	A full description of the statistical parameters including central tendency (e.g. means) or other basic estimates (e.g. regression coefficient) AND variation (e.g. standard deviation) or associated estimates of uncertainty (e.g. confidence intervals)
☐	☒	For null hypothesis testing, the test statistic (e.g. F, t, r) with confidence intervals, effect sizes, degrees of freedom and P value noted<br>*Give P values as exact values whenever suitable.*
☒	☐	For Bayesian analysis, information on the choice of priors and Markov chain Monte Carlo settings
☒	☐	For hierarchical and complex designs, identification of the appropriate level for tests and full reporting of outcomes
☒	☐	Estimates of effect sizes (e.g. Cohen's d, Pearson's r), indicating how they were calculated

Our web collection on statistics for biologists contains articles on many of the points above.

Software and code

Policy information about availability of computer code

Data collection	Images were acquired using Metamorph (Molecular Devices), Zen 2012 (Zeiss) and NimOS (ONI).
Data analysis	Images were analyzed using ImageJ 1.5j8 (NIH), Zen 3.5 (Blue Edition; Zeiss) and MATLAB 2017b. Numerical data were analyzed using MATLAB R2017b (Mathworks), R Studio (R Project) and Prism 9 (Graphpad).<br><br>Particle detection and tracking was performed using u-track, https://github.com/DanuserLab/u-track (https://doi:10.1038/nmeth.1237).<br><br>Particle trapping was analyzed using Transient Trapping Analysis, https://github.com/YannLanoiselee/Transient_trapping_analysis (https://doi.org/10.3390/e23081044.<br><br>Particle clustering was analyzed using DBSCAN, implemented in the ClustDoC package (https://github.com/PRNicovich/ClusDoC/tree/Clus-DoC/private).

For manuscripts utilizing custom algorithms or software that are central to the research but not yet described in published literature, software must be made available to editors and reviewers. We strongly encourage code deposition in a community repository (e.g. GitHub). See the Nature Portfolio guidelines for submitting code & software for further information.

Data

Policy information about availability of data

All manuscripts must include a data availability statement. This statement should provide the following information, where applicable:

- Accession codes, unique identifiers, or web links for publicly available datasets
- A description of any restrictions on data availability
- For clinical datasets or third party data, please ensure that the statement adheres to our policy

All data extracted from the raw image files is provided in the source data file. Source data are provided with this paper.

Human research participants

Policy information about studies involving human research participants and Sex and Gender in Research.

Reporting on sex and gender	Not applcable.
Population characteristics	Not applicable.
Recruitment	Not applicable.
Ethics oversight	Not applicable.

Note that full information on the approval of the study protocol must also be provided in the manuscript.

Field-specific reporting

Please select the one below that is the best fit for your research. If you are not sure, read the appropriate sections before making your selection.

[x] Life sciences [ ] Behavioural & social sciences [ ] Ecological, evolutionary & environmental sciences

For a reference copy of the document with all sections, see nature.com/documents/nr-reporting-summary-flat.pdf

Life sciences study design

All studies must disclose on these points even when the disclosure is negative.

Sample size	The measurement unit is animal, batch of islets or well of cells. Experiments were repeated independently at least three times, usually with technical replicates. Islet data are reported from at least three separate isolation procedures. Effect size was determined from pilot studies as well as similar experiments in the published literature. A priori sample size calculations were performed in G*Power 3.1.9.2 based upon the expected effect size (d), using a test power = 0.9, alpha = 0.05 and the difference between two independent means.
Data exclusions	No data were excluded unless the cells displayed a clear non-physiological state (i.e. impaired viability).
Replication	All findings were replicated independently at least three times.
Randomization	Samples and animals were allocated to treatment groups in a randomized manner to ensure that all states were represented in the different experiment arms.
Blinding	Data were acquired using imaging setups that performed the measurement independently of the observer. For non-imaging experiments, i.e. in vivo experiments, investigators were blinded to animal genotype.

Reporting for specific materials, systems and methods

We require information from authors about some types of materials, experimental systems and methods used in many studies. Here, indicate whether each material, system or method listed is relevant to your study. If you are not sure if a list item applies to your research, read the appropriate section before selecting a response.

Materials & experimental systems

n/a	Involved in the study
[ ]	[x] Antibodies
[ ]	[x] Eukaryotic cell lines
[x]	[ ] Palaeontology and archaeology
[ ]	[x] Animals and other organisms
[x]	[ ] Clinical data
[x]	[ ] Dual use research of concern

Methods

n/a	Involved in the study
[x]	[ ] ChIP-seq
[x]	[ ] Flow cytometry
[x]	[ ] MRI-based neuroimaging

Antibodies

Antibodies used	GLP1R Iowa DSHB Cat# Mab 7F38, RRID:AB_2618101.<br>Goat anti-mouse DyLight488 Thermo Fisher Scientific Cat #35502, RRID:AB_844397.
Validation	GLP1R Mab was previously validated by us and others using tissue from GLP1R-/- mice (10.1210/en.2017-00812 and 10.1038/s41467-020-14309-w).

Eukaryotic cell lines

Policy information about cell lines and Sex and Gender in Research

Cell line source(s)	AD293 (Agilent)
Authentication	AD293 cells were authenticated using STR profiling against the related HEK293T cell line.
Mycoplasma contamination	Mycoplasma testing was carried out routinely every three months, with all results negative.
Commonly misidentified lines (See ICLAC register)	No commonly misidentified cell line was used in the study.

Animals and other research organisms

Policy information about studies involving animals; ARRIVE guidelines recommended for reporting animal research, and Sex and Gender in Research

Laboratory animals	GLP1R-SNAP/SNAP knock-in mice, generated using CRISPR. Homozygous and wild-type littermates were used in a number of experiments.<br><br>Gt(ROSA)26Sortm1.1(CAG-cas9*,–EGFP)Fezh/J (JAX stock no. 024858) mice were used as embryo donors for generation of GLP1R-SNAP/SNAP knock-in mice.<br><br>All animals were male and female, 6-12 wks.<br><br>Mice were socially-housed in specific-pathogen free conditions under a 12 hour light-dark cycle with ad libitum access to food and water, relative humidity 55 ± 10% and temperature 21 ± 2 °C.
Wild animals	No wild animals were used in the present study.
Reporting on sex	To exclude any sex-specific effects of GLP1R-SNAP/SNAP knock-in, male and female animals were considered separately for metabolic phenotyping experiments. All in vitro studies combined tissue from both sexes, since: 1) no sex-specific phenotype was observed in vivo; and 2) male and female tissues were indistinguishable by their SNAP-tag labelling or GLP1R staining. For glucose tolerance and body weights, each sex was analyzed separately versus their wild-type controls using two-way repeated measures ANOVA.
Field-collected samples	No field-collected samples were used in the present study
Ethics oversight	Animal studies were regulated by the Animals (Scientific Procedures) Act 1986 of the U.K. (Personal Project Licences P2ABC3A83 and PP1778740). Approval was granted by the University of Birmingham's Animal Welfare and Ethical Review Body (AWERB). All ethical guidelines were adhered to whilst carrying out this study.

Note that full information on the approval of the study protocol must also be provided in the manuscript.
